# Cultural factors related to childhood and adolescent obesity in Mexico: A systematic review of qualitative studies

**DOI:** 10.1111/obr.13461

**Published:** 2022-05-19

**Authors:** Magaly Aceves‐Martins, Lizet López‐Cruz, Marcela García‐Botello, Naara L. Godina‐Flores, Yareni Yunuen Gutierrez‐Gómez, Carlos Francisco Moreno‐García

**Affiliations:** ^1^ The Rowett Institute of Nutrition and Health University of Aberdeen Aberdeen UK; ^2^ Universidad Europea del Atlantico Parque Cientifico y Tecnologico de Cantabria Santander Spain; ^3^ Universidad de Monterrey San Pedro Garza García Mexico; ^4^ Nutrition Department, School of Medicine and Health Sciences Tecnológico de Monterrey Mexico City Mexico; ^5^ School of Medicine and Health Sciences Tecnologico de Monterrey Zapopan Mexico; ^6^ School of Computing Robert Gordon University Aberdeen UK

**Keywords:** adolescents, children, cultural factors, Mexico, obesity, qualitative

## Abstract

Culture and culturally specific beliefs or practices may influence perceptions and decisions, potentially contributing to childhood obesity. The objective of this study is to identify the cultural factors (expressed through decisions, behaviors, individual experiences, perceptions, attitudes, or views) related to childhood and adolescent obesity in Mexico. Ten databases and one search engine were searched from 1995 onwards for qualitative studies. The Sunrise Enabler Model, described within the Cultural Care Theory, guided this review. Sample, the phenomenon of interest, study design, and evaluation data were extracted, and the Critical Appraisals Skills Programme tool was used to assess the quality of the included studies. Twenty‐four studies were included. Of these, 12 studies included children or adolescents, 12 included parents, eight included schoolteachers, four included school staff (other than teachers), four included food vendors, and one included policymakers. Cultural values, beliefs, lifeways (especially food and food costumes), kinship, and social factors (particularly immediate and extended family) strongly influenced childhood and adolescent obesity‐related lifestyles in Mexico. Most cultural factors related to childhood obesity in Mexico identified in this review may be modifiable and amenable to practical interventions.

## 1 INTRODUCTION

The complex relation of behavioral, sociocultural, economic, and environmental factors has been described in the literature as risk factors for developing childhood or adolescent obesity.^1^, ^2^, ^3^ In addition, evidence from ethnic minorities in high‐income countries has described that culture and culturally specific beliefs or practices may influence strategies, perceptions, and decisions (from children, caregivers, or other stakeholders).^4^, ^5^, ^6^, ^7^ As a result, children's and adolescents' health status and behaviors are shaped differently, potentially contributing to obesity.^4^, ^5^ For instance, feeding practices, preferences, health and food‐related beliefs, or cultural influences have been described as potential contributors to the increased risk of obesity among Latinos/Hispanos children and youth in the United States.^4^, ^5^

Qualitative research explores how people perceive and experience certain phenomena. This type of research typically relies on interviews or observations that explore people's perceptions, beliefs, practices, and experiences in connection with their health or health care services use.^8^ There can be an increased understanding of a specific phenomenon within a specific context by synthesizing qualitative evidence. Moreover, associations between broader environments and understanding the values, attitudes, and experiences of health conditions and interventions can also be achieved through the synthesis of qualitative literature.^9^ Some research has been done on determining factors relevant to childhood obesity through qualitative research synthesis.^3^ A previous paper by Chatham and Mixer^3^ synthesized qualitative evidence of obesity‐promoting factors in ethnic minorities across the United States. Such work added great value to previous research on cultural factors of obesity among Mexican‐origin children or adolescents as an ethnic minority in the United States.^3^, ^4^, ^5^ Nonetheless, the factors identified so far among Mexican‐origin children and adolescents might differ from those decisions, behaviors, individual experiences, perceptions, attitudes or views in children or adolescents living in Mexico because migration and acculturation might shape some health behaviors.^5^, ^10^

Obesity rates in Mexico have been alarmingly increasing in the last decades.^11^ Such rates have been notoriously high among the < 18 years old population, where it is estimated that by 2018 over 8% of infants (0–4 years), 35% of school‐age children (5–11 years), and almost 40% of adolescents (12–19 years) had overweight or obesity.^12^ Furthermore, interventions to either prevent^13^ or treat^14^ obesity among Mexican children and/or adolescents rarely consider cultural factors and focus merely on behavioral change among children and adolescents. Therefore, identifying cultural factors related to obesity among childhood and adolescent within the Mexican culture is indispensable to tackling it effectively. The “Childhood and adolescent Obesity in MexicO: evidence, challenges and opportunities” (COMO) Project intends to synthesize and use data to comprehend the extent, nature, effects, and costs of childhood and adolescent obesity in Mexico.^13^, ^14^, ^15^, ^16^ This review of qualitative studies is part of the COMO project and aims to identify the cultural factors (expressed through decisions, behaviors, individual experiences, perceptions, attitudes, or views) related to childhood and adolescent obesity in Mexico.

## 2 METHODS

This project's systematic review was registered in The International Prospective Register of Systematic Reviews (PROSPERO Registration number CRD42019154132),^17^ and it is reported according to Preferred Reporting Items for Systematic Reviews and Meta‐analyses (PRISMA) guidelines.^18^ The research question and inclusion/exclusion criteria were defined following the Sample, Phenomenon of Interest, Design, Evaluation, Research type (SPIDER) framework for qualitative synthesis.^19^

A sensitive search was developed to include index terms, free‐text words, abbreviations, and synonyms to combine the key concepts for this review (Table S1). The databases searched included MEDLINE, EMBASE, Global Health Library, LILACS, CINAHL, ERIC, PsycINFO, ScienceDirect, Scopus, AGRICOLA, and SciELO Citation Index. When possible, searches were also done in Spanish to capture relevant references. In addition, the search engine Google Scholar and the COMO project database were also searched. The COMO project database comprises over 950 references related to childhood and adolescent obesity in Mexico.^15^

In addition, reference lists of included papers were scrutinized for additional publications. Abstracts were excluded from this review. Studies published from 1995 onwards were considered in this review. Original searches were done in January 2020 and updated in January 2022.

### 2.1 Selection criteria

The eligibility criteria were based on the SPIDER framework:

*Sample*: Studies that included (or referred to) children and adolescents ≤18 years old from any ethnicity living in Mexico were considered. Studies analyzing data on children with severe conditions (e.g., cancer, HIV, and Down syndrome) or pregnant adolescents were excluded. Also, studies of Mexican children living in a different country were excluded to avoid information inherent to the migration phenomena and acculturation.

Studies that included data from direct caregivers of children or adolescents (e.g., parents, teachers, or health professionals), indirect caregivers (e.g., school principals), and/or relevant stakeholders on childhood obesity matters (e.g., school food vendors or policymakers) were also included if the study aimed to comprehend their views and beliefs regarding childhood obesity in Mexico.

Phenomenon of Interest
: : Childhood and adolescent obesity in the Mexican context.
Design
: : Studies using any qualitative design, following any framework or theory, were included.
Evaluation
: : Cultural factors (expressed through decisions, behaviors, individual experiences, perceptions, attitudes, or views) reported through quotes from participants and/or interpretation of findings by study authors.
Research type
: : Any qualitative or mixed methods studies were considered. However, mixed methods studies were included only if the qualitative methods and results were reported separately from the quantitative analysis.

### 2.2 Conceptual framework

This systematic review was conducted following the Sunrise's Cultural and Social Structure Dimensions Enabler Model described within Leininger's theory of culture care diversity and universality, also known as the cultural care theory (CCT).^20^, ^21^, ^22^ The Sunrise's Cultural and Social Structure Dimensions Enabler Model is a cognitive guide of the theory used to guide our culture care phenomena from a holistic perspective of the multiple factors shaping the well‐being of diverse cultures.^22^ Such a model illustrates areas that need to be explored regarding the CCT theory principles.^22^ Similar to those described by the CDC^23^ and the Socio‐Ecological Model,^24^, ^25^ Sunrise's Cultural and Social Structure Dimensions Enabler Model also considers the social structure dimensions of health constructs at different levels (e.g., individual, interpersonal, community, or policy level).

To reflect on the Mexican culture's dynamic, holistic, and interrelated patterns, eight cultural and social structure dimensions from the Sunrise's Cultural and Social Structure Dimensions Enabler Model were considered: (1) Biological factors; (2) Cultural values, beliefs, lifeways; (3) Economic factors; (4) Education factors; (5) Kinship and social factors; (6) Political and legal factors; (7) Religious and philosophical factors, and (8) Technological factors (details on each of these dimensions are provided in Table S2).

### 2.3 Data selection and extraction

Titles, abstracts, and relevant full texts were screened by three reviewers (LL, MGB, MA‐M). In addition, two reviewers (MA‐M and LL) independently extracted data from relevant studies. A data extraction form was developed based on the CCT theory principles^22^ and piloted for this systematic review. From each included study, we recorded quotes from participants and/or interpretation of findings by study authors irrespective of whether participants' quotes supported it. Besides the free codes identified, the participants/stakeholder role was also recorded: Individual (i.e., quotes from children or adolescents); Interpersonal (e.g., quotes from direct caregivers, such as parents and teachers); Community, Institutional, or Industry (i.e., quotes from school principals or school food vendors); Policy (i.e., quotes from policymakers or academics). Papers were initially organized alphabetically and subsequently grouped under themes.

### 2.4 Data analysis

A thematic synthesis using both inductive and deductive approaches was done. First, to identify the main recurring themes, reviewers conducted a line‐by‐line coding of the qualitative findings of each of the included studies. Next, guided by the Sunrise's Cultural and Social Structure Dimensions Enabler Model,^20^, ^21^, ^22^ “free codes” (i.e., single quotes) were organized into related areas to construct “descriptive themes”, then organized into “analytical themes.” If appropriate, free codes were recorded in more than one descriptive or analytical theme. Finally, codes from studies in Spanish were translated to English using the back‐translation method.^26^ A bilingual reviewer (MA‐M) translated the codes into English, and a different bilingual reviewer (CMF‐G) translated the material back into Spanish to test the accuracy of the translation.

Results were discussed among five reviewers (MA‐M, LLC, NGL, YYGG, and MGB) to ensure consistency across codes and their designations to different themes. Results are reported narratively, and the main results are also tabulated. Analytic and descriptive themes are described in this review based on code density.

### 2.5 Quality assessment

Methodological rigor and theoretical relevance of included studies were appraised through the Critical Appraisals Skills Programme (CASP) tool,^27^ recommended for quality appraisal in qualitative evidence synthesis. CASP appraises the strengths and limitations through questions that focus on different methodological aspects of a qualitative study, such as clarity of the aim, methods appropriateness, or data collection. Included studies were quality‐appraised independently by two reviewers (MAM and LLC). Any disagreement was resolved by discussion with a third reviewer (YYGG).

## 3 RESULTS

**FIGURE 1:**
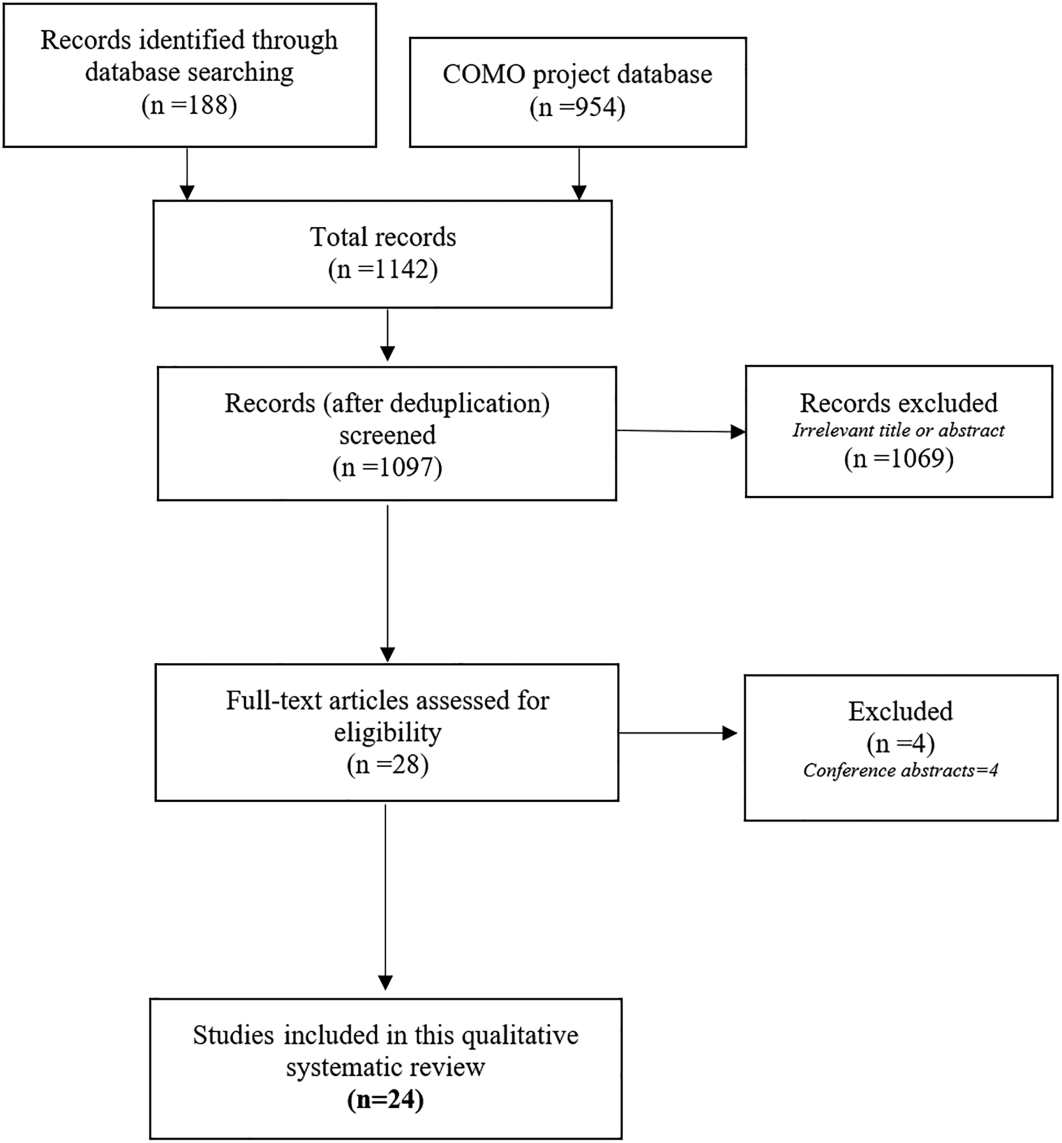
Preferred Reporting Items for Systematic Reviews and Meta‐analyses (PRISMA) flow diagram

**FIGURE 2:**
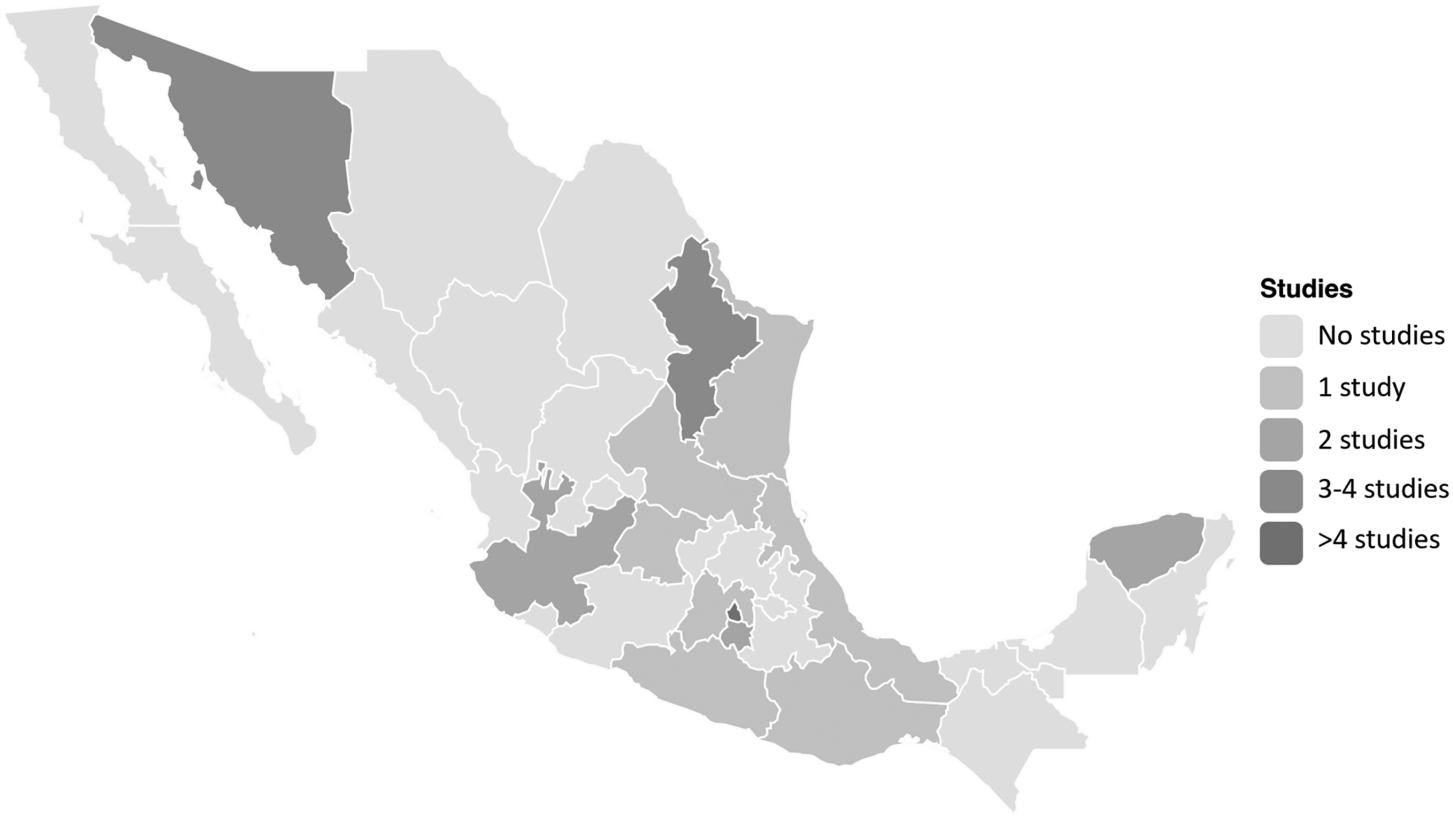
Map of the evidence

After searching the different databases, 1097 references were identified, of which 28 were retrieved for full‐text review. Of these, 24 studies^28^, ^29^, ^30^, ^31^, ^32^, ^33^, ^34^, ^35^, ^36^, ^37^, ^38^, ^39^, ^40^, ^41^, ^42^, ^43^, ^44^, ^45^, ^46^, ^47^, ^48^, ^49^, ^50^, ^51^ met the inclusion criteria for this review (Figure 1). Studies were conducted in 16 out of 32 Mexican federal states (Figure 2). Two studies^31^, ^37^ were conducted in more than one Mexican state. All the included studies were published during or after 2010. Overall, 12 studies^33^, ^35^, ^36^, ^38^, ^39^, ^40^, ^44^, ^45^, ^46^, ^48^, ^49^, ^51^ included children or adolescents, 12 included parents,^29^, ^30^, ^32^, ^34^, ^35^, ^36^, ^37^, ^41^, ^42^, ^47^, ^49^, ^51^ eight included teachers,^28^, ^30^, ^31^, ^35^, ^36^, ^43^, ^49^, ^51^ four included school principals or other school staff (not directly in charge of the child's care),^28^, ^30^, ^43^, ^49^ four included school food vendors or school's kitchen staff,^28^, ^30^, ^43^, ^49^ and one included policymakers or academics (Table A1).^50^ Most of the studies were qualitative and used structured or semi‐structured interviews with thematic analysis.

**FIGURE 3:**
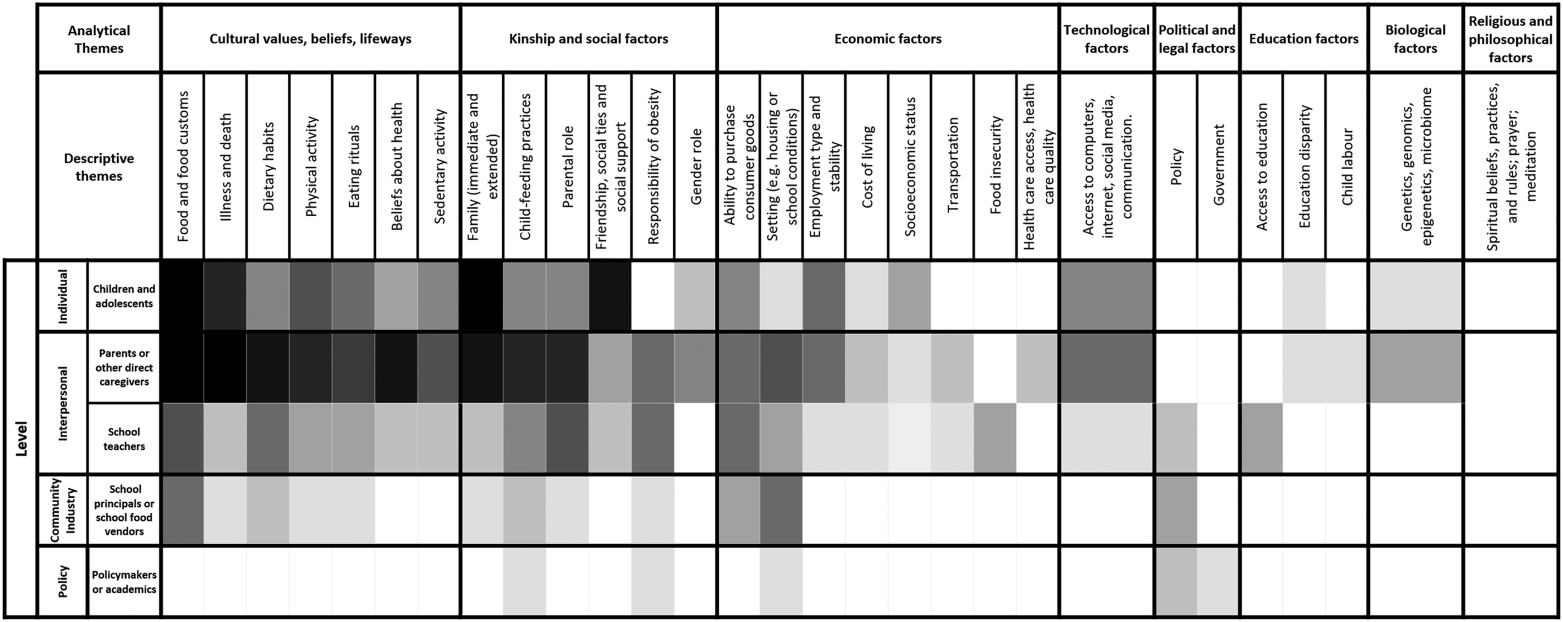
Heatmap of identified themes

Code density of descriptive and analytical themes is presented in Figure 3. The results presented in this section are ordered according to the density of the themes. Those themes with a higher density are presented first. Only those with the higher densities found in more than one study are described in the following section. The complementary list of the themes (including those with a lower density and only presented in one study) can be consulted in Appendix B. In addition, some examples of free codes are presented in Table 1.

**TABLE 1:** Descriptive and analytical themes identified

Analytical themes	Descriptive themes	Level of identified free codes				Level and code example
		Individual	Interpersonal	Community Industry	Policymakers	
Cultural values, beliefs, lifeways	Food and food customs	✓	✓	✓	×	Individual level: “… the food is the most delicious thing at my house, I like when my mom prepares breaded [foods] is what I like the most …” (adolescent from a public school).^44^ ^,^ ^a^ Interpersonal level: “Things taste better with Coke,” “He [referring to her son] is happy when he drinks soda and fruit drinks” (mothers from a young child attending kinder in a marginalized area).^41^
	Illness and death	✓	Parents: ✓ Teachers: ×	✓	×	Interpersonal level: “My husband is diabetic, and they have told us that my son is overweight and that we should avoid breaded and fried foods” (mother accompanying a preschool child with overweight at a public clinic receiving preventive or curative health care for problems unrelated to weight).^33^
	Dietary habits	✓	Parents: ✓ Teachers: ✓	✓	×	Interpersonal level: “He [referring to the children] eats with me after school, but then he eats with his grandparents that live next door, and if the other neighbour offers him food, he'll eat again” (parent of a child with obesity).^40^
	Physical activity	✓	Parents: ✓ Teacher: ✓	✓	×	Individual level: “Exercise helps because when we sweat, we get all the fat [out]” (12‐year‐old participant from a rural school).^45^ ^,^ ^a^
	Eating rituals	✓	Parents: ✓ Teacher: ✓	✓	×	Individual level: “Almost every day [I get a snack as a reward]” (girl from an urban school at a low SES area, no further characteristics provided).^51^ Interpersonal level: “If we have a meal that we really enjoy, for example, enchiladas or something similar, then we feel it deserves to be served with soda” (mother from a young child attending kinder in a marginalized area).^41^
	Beliefs about health	✓	Parents: ✓ Teacher: ✓	×	×	Interpersonal level: “They have told me that my son is obese, but I am not very worried about it – as they grow their weight goes down …” (mother accompanying a preschool child with overweight at a public clinic receiving preventive or curative health care for problems unrelated to weight).^34^
	Sedentary activity	✓	Parents: ✓ Teacher: ✓	×	×	Interpersonal level: “… children hardly practice sports [they prefer] to spend their free time [doing other activities] I do not know, watching television, playing games” (physical education teacher from a public secondary school).^28^ ^,^ ^a^
Kinship and social factors	Family	✓	Parents: ✓ Teacher: ✓	✓	×	Individual level: “I ask [money to] my granny hahaha, and she gives me more money [for buying junk food] than my parents” (adolescent from a public school).^46^ ^,^ ^a^
	Child‐feeding practices	✓	Parents: ✓ Teacher: ✓	✓	✓	Individual level: “Sometimes my mother does not have time to make us breakfast, and then we eat something from the supermarket …” (student from a public school of an urban area).^33^
	Parental role	✓	Parents: ✓ Teacher: ✓	✓	×	Individual level: “… [we have as breakfast] whatever my mom prepares” (Adolescent from a public school).^38^ ^,^ ^a^ “… My dad always takes me to the park on Wednesdays … there I play basketball” (child from a public school in an urban area).^48^ ^,^ ^a^
	Responsibility of obesity	×	Parents: ✓ Teacher: ✓	✓	✓	Industry level: “[Parents] became negotiators with their children *…* [referring to dietary behaviors and healthy lifestyles]” (executive at the Mexican Council of the Consumer Products Industry).^50^ ^,^ ^a^
	Friendship, social ties and social support	✓	Parents: ✓ Teacher: ✓	×	×	Individual level: “If they make fun of my fat, I will not talk to them again or stop playing with them, because that means they are not my friends” (child with obesity, no further details provided).^38^ ^,^ ^a^
	Gender role	✓	Parents: ✓ Teacher: ×	×	×	Individual level: “I say that a boy should be bigger than a girl …” (female adolescent, with normal weight from a public secondary school).^39^ “… my mother tells me that I am fat and that I should take care of myself because I am a woman” (child with obesity, no further details provided).^38^ ^,^ ^a^
Economic factors	Ability to purchase consumer goods	✓	Parents: ✓ Teacher: ✓	✓	×	Interpersonal level: “We shop daily […] I cannot buy like that [weekly], I can't go to a supermarket or go to the market and bring all week supplies” (mother from a young child attending kinder in a marginalized area).^41^ Community level: “… There are [street vendors outside the school] who sell pork crackers, and all what they [students] like: junk food …” (head of the school store of a public secondary school).^28^ ^,^ ^a^
	Setting	✓	Parents: ✓ Teacher: ✓	✓	✓	Individual level: “[when asked if the participant can go to the park alone] No, because it is not a safe place for unsupervised children” (girl from an urban school at a low SES area, no further characteristics provided).^51^
	Employment type and stability	✓	Parents: ✓ Teacher: ✓	×	×	Individual level: “My mother works and rests only on Mondays, she does not cook [the food], my sister is the one who does it” (student from a public school of an urban area, no further characteristics provided).^33^
	Cost of living	✓	Parents: ✓ Teacher: ✓	×	×	Interpersonal level: “The paediatrician put him on a diet, but then I do not keep the diet because I do not have the [economic] means to follow it …” (mother accompanying a preschool child with overweight at a public clinic receiving preventive or curative health care for problems unrelated to weight).^34^
	Socioeconomic status	✓	Parents: ✓ Teacher: ×	×	×	Individual level: “Guadalupe's construction of public spaces as unsafe [referring to crime and public insecurity in low socioeconomic areas] illustrates the challenges many children face living in low socioeconomic areas in the [capital] city” (authors' observation).^51^
	Transportation	×	Parents: ✓ Teacher: ✓	×	×	Interpersonal level: “Time constraints … attributed to the heavy traffic in Mexico City, which makes commuting lengthy … [hence, reducing drastically the time to cook at home or exercise with the family children]” (authors' observation).^51^
	Food insecurity	×	Parents: × Teacher: ✓	×	×	Interpersonal level: “… There are children who come without breakfast, a little while ago a boy approached me to rate him and told me ‐ oh my tummy is rumbling …” (teacher from a rural and indigenous school).^31^ ^,^ ^a^
	Health care access & health care quality	×	Parents: ✓ Teacher: ×	×	×	Interpersonal level: “When I bring him in for vaccinations, they scold me, ‘why is your son so overweight? He's very overweight … you have to bring him to nutrition and put him on a diet’” (mother accompanying a preschool child with overweight at a public clinic receiving preventive or curative health care for problems unrelated to weight).^34^
Technological factors	Access to computers, the internet or social media	✓	Parents: ✓ Teacher: ✓	×	×	Interpersonal level: “… I am a worker, and as long as the children are indoors and not outside, I pay cable TV so that they can watch it … [making emphasis on how sedentary lifestyles are unintentionally promoted]” (mother from a preschool child).^36^ ^,^ ^a^
Political and legal factors	Policy	×	Parents: × Teacher: ✓	✓	✓	Community level: “They just gave me what the recipe is, [this is the list of] the products that I could sell or not, and it was from the management” (head of the school store of a public secondary school).^28^ ^,^ ^a^
	Government	×	×	✓	✓	Policy level: “[childhood obesity rates are] violation of this right to health by omission. [enphasising that policymakers should also be acknowledged as relevant stakeholders in childhood obesity matters]” (head of a non‐governmental organization for defending consumer rights).^50^ ^,^ ^a^
Education factors	Access to education	×	Parents: × Teacher: ✓	×	×	Interpersonal level: “Is not as good as counselling or as something specific, but it is generally commented [about nutrition] in each of the meetings we have with teaching staff” (teacher from a public secondary school).^28^ ^,^ ^a^
	Education disparity	✓	Parents: ✓ Teacher: ×	×	×	Individual level: “He [referring to a school peer with obesity] has bad grades because they do not put him in good teams because here we work almost all the time as a team [and as a result of having obesity, he is placed in worst teams]” (female adolescent, with normal weight from a public secondary school).^39^
	Child labor	×	Parents: × Teacher: ✓	×	×	Interpersonal level: “[Referring to children from low SES] The daily subsistence requires the work of both parents and, sometimes, of the children [which are vulnerable to not fulfilling their nutritional requirements]” (authors' observation).^31^
Biological factors	Genetics, genomics, epigenetics, microbiome	✓	Parents: ✓ Teacher: ×	×	×	Individual level: “It's normal that I'm fat, my family is fat” (adolescent with obesity from a public school).^40^

^a^ Codes translated from Spanish to English. ✓ Free codes identified at this level. × No free codes identified at this level. SES = socioeconomic status.

### 3.1 Cultural values, beliefs, lifeways

#### 3.1.1 Food and food customs

Across all levels (i.e., individual, interpersonal, community/industry, or policy), food and food customs were the descriptive themes most frequently associated with childhood or adolescent obesity. Overall, the conception of a good diet was limited to consuming fruits and vegetables.^30^, ^33^, ^35^, ^38^, ^41^, ^45^, ^46^, ^48^, ^49^ In the narratives, the term “junk food” usually refers to ultra‐processed products with high fat, salt, and/or sugar content (e.g., crisps or candies) and were considered “unhealthy products.”^33^, ^35^, ^36^, ^42^, ^43^ Nevertheless, some discrepancies exist when identifying “junk” products across free codes. For instance, sodas are considered “junk” or “unhealthy products,” but most drinks (including sweetened juices and sports or sugary drinks but not sodas) were considered “healthy” by children, parents, and teachers.^33^, ^35^, ^36^, ^41^, ^42^, ^43^ Also, parents often categorize cereals or starchy carbohydrates as “unhealthy,” including “tortillas,” made of cornflour and the Mexican cuisine base.^33^, ^34^, ^35^, ^36^, ^37^, ^42^, ^47^, ^49^

Children and some parents associate food's quantity (rather than quality) with obesity.^32^, ^46^, ^49^ Additionally, children prioritize food's flavor over its nutritional value.^30^, ^32^, ^33^, ^35^, ^38^, ^46^, ^48^, ^49^, ^51^ The relevance of taste in the children's food decisions was also highlighted by several parents,^33^, ^34^, ^35^, ^36^ school staff, and food vendors.^28^, ^30^, ^43^, ^49^ Most of the food choices children make at school are mainly based on food availability and taste.^30^, ^32^, ^33^, ^35^, ^38^, ^46^, ^48^, ^49^, ^51^ Overall, parents and teachers described food available in schools as “junk” or “unhealthy.”^30^, ^33^, ^35^, ^38^, ^45^, ^46^, ^48^, ^49^ School food vendors interviewed in one study^30^ emphasized their main purpose was to offer hygienic foods and please the children's preferences rather than consider their nutritional needs. Likewise, food vendors believed that fruits and vegetables were not suitable to sell, mainly because the preparation is time demanding (e.g., peeling and cutting) and have a limited shelf life compared with other products.

Some children reported being susceptible to food marketing campaigns, and some parents also acknowledge such susceptibility among children.^39^, ^46^, ^48^, ^49^ Parents also trust marketing campaigns or slogans (e.g., “light”), which made them believe products were “healthy” options and could aid obesity prevention.^34^, ^42^, ^43^, ^49^, ^50^ Additionally, one study reported that one international soda company had provided resources to reform the school. Hence, teachers at the school promoted the consumption of such products among students to get further benefits from the company.^31^

#### 3.1.2 Illness and death

Overall, childhood and adolescent obesity in Mexico was perceived as an esthetic issue, not a health problem. Most children were unaware of their weight status and the detrimental short‐ and long‐term health issues associated with overweight or obesity.^32^ Children only reflected health issues related to obesity with adult relatives, not with themselves or other children.^39^, ^40^, ^45^, ^46^, ^48^, ^49^ Some children and adolescents with obesity accept having physical difficulties doing everyday tasks (e.g., agitation or lack of breath). However, these are not considered health issues.^39^, ^40^ Some children with obesity did not feel physically bad but emotionally sad because of their appearance or for not fitting in the clothes they liked.^32^, ^39^, ^40^, ^46^ Remarkably, several children with obesity show a lack of interest or worry when their weight issue is brought to their attention and seem reluctant to a lifestyle improvement.^30^, ^32^, ^38^, ^40^, ^44^

Parents limited childhood obesity to an esthetic problem and did not consider their children's weight status as a reason to seek medical advice.^29^, ^30^, ^32^, ^34^, ^35^, ^37^, ^41^, ^42^, ^47^ Most codes from parents showed an unconcern for the risks associated with childhood obesity and appeared to excuse the obesogenic behaviors of their children.^29^, ^30^, ^32^, ^34^, ^35^, ^37^, ^42^, ^47^, ^51^ Some parents showed concerns about tooth decay in children with obesity but not to potential comorbidities related to obesity.^34^, ^47^, ^49^ Teachers and school principals also normalized overweight among students.^30^, ^35^

#### 3.1.3 Dietary habits

Participants acknowledge a gastronomic transition, and most believe this is an obesity risk factor. Homemade foods are given more value and are believed to be a “healthier” option.^33^, ^35^, ^37^, ^38^, ^46^, ^48^, ^49^, ^51^ However, most participants consider some foods sold at school canteens or street food businesses as “homemade type,” hence “healthy.”^30^, ^32^, ^35^, ^46^, ^48^, ^49^, ^50^ Traditional dishes (e.g., “tacos” or “enchiladas”) seem to be valued by children and parents and are considered an ideal option to be provided as family meals.^30^, ^33^, ^41^, ^46^, ^49^, ^51^ Only a few parents noticed that traditional dishes are not always the “healthiest” option, especially if eaten away from home.^37^, ^42^

No children associated cooking methods with a higher risk of obesity. However, parents and teachers acknowledged that some cooking methods (e.g., steamed) might be “healthier” than others (e.g., fried). Even though cooking methods such as breaded or fried are considered “unhealthy,” mothers report using them because it is the only way children accept to eat certain foods (e.g., vegetables or fish).^30^, ^33^, ^34^, ^35^, ^36^, ^42^, ^43^, ^47^, ^49^ In addition, mothers perceive in children selectiveness and resistance towards certain foods (e.g., vegetables), and if served at home, some feel they are providing their children with foods they dislike.^33^, ^35^, ^36^, ^43^

Children, parents, and teachers recognize sugary drinks as part of the children's daily diets and are often associated with happiness or as a comfort beverage.^35^, ^41^, ^42^, ^46^, ^48^, ^49^, ^51^ Some parents and teachers identify sugary drinks as “unhealthy”; however, they kept serving them for acceptability with children.^32^, ^41^, ^43^ In most narratives, traditional Mexican dishes were usually complemented with soft drinks.^35^, ^41^, ^42^, ^46^, ^48^, ^49^, ^51^ Some parents try to limit these drinks at home but report that children are highly exposed to them in out‐of‐home environments.^32^, ^34^, ^41^, ^42^

Children and mothers acknowledge the importance of different food meals throughout the day (e.g., breakfast, lunch, and dinner). However, in most of the studies, it was highlighted that children have several opportunities to eat inside the schools (e.g., school canteens), outside the schools (e.g., street food vendors), and at home (or relative's homes) in a single day.^35^, ^36^, ^38^, ^46^ However, only parents recognized this as a potentially “unhealthy” lifestyle leading to obesity.^32^, ^33^, ^42^, ^43^ Children recurrently buy food at school, after school, or other places rather than eating at home or homemade items.^30^, ^33^, ^38^, ^51^ Most children preferred bringing money to school rather than bringing homemade lunches.^30^, ^33^, ^38^, ^51^ Most parents report giving money to their children and recognize that they usually make poor decisions while buying food.^30^, ^33^, ^34^, ^35^, ^36^, ^48^, ^49^ Teachers also reported children having money during school hours as a significant barrier to convincing them to eat healthier.^43^

Children (as young as 6 years old) have autonomy in their dietary choices, including what they eat at home and outside the home.^30^, ^33^, ^38^, ^51^ However, most parents think they do not have enough control over what children eat during school hours, and children's preferences define food and drinks provided at home.^30^, ^33^, ^34^, ^35^, ^36^, ^47^

#### 3.1.4 Physical activity

Overall, children consider physical activity a weight management strategy rather than a recreational activity or healthy lifestyle.^36^, ^38^, ^39^, ^40^, ^45^ Children perceive that those peers with obesity might experience some difficulties when exercising because of the excess weight.^39^, ^40^, ^45^ Parents also consider physical activity a weight management strategy,^29^, ^34^, ^42^, ^47^ and some mentioned challenges while trying to perform physical activities with their children (e.g., children's dislike, costs, safety, or lack of time).^29^, ^34^, ^42^, ^43^, ^47^, ^51^

Teachers and health professionals at schools acknowledge the importance of physical activity in obesity prevention.^28^, ^36^, ^43^ Most report not having a structured physical education class or lacking knowledge on motivating children to engage effectively in physical activities.^28^, ^36^, ^43^

#### 3.1.5 Eating rituals

Overall, across narratives, food is a tradable currency that rewards or punishes children for certain behaviors, controlling the parents' and children's relationship. Such rewards were reported as products that children value but have high fat, salt, or sugar content (e.g., crisps or candies).^30^, ^34^, ^36^, ^42^, ^43^, ^47^, ^51^ Rewarding children with food was also reported by teachers.^30^

Some parents reported using family dinner time to update the head of the household (typically fathers) on the child's behavior during the day.^35^ Likewise, parents expect no leftovers on the plates; even if children are not hungry, they need to finish all the portion that has been served.^36^, ^37^

#### 3.1.6 Beliefs about health

At an interpersonal level, parents and extended family (e.g., grandmothers) believe that a “chubby child” is a healthy child.^29^, ^30^, ^34^, ^37^, ^42^ Some believe that children will undergo a “growth spurt” after a weight gain or might have “big‐bones.”^29^, ^34^, ^37^, ^42^ Some mothers believe children should eat more than adults because “they use more energy.”^32^, ^37^ As a result, parents and extended family (e.g., grandmothers) underestimate childhood obesity.^29^, ^32^, ^34^, ^42^, ^47^ Some recognized obesity as a disease (only in adults) and expressed more concerns about avoiding nutrient deficiencies and being underweight among children than having overweight.^42^, ^47^ Across all the free codes identified, a pattern was seen where mothers used synonyms with positive connotations when referring to children with obesity (e.g., “he is gaining a little weight,” “he is robust, not fat,” and “she is a bit chubby”).^29^, ^32^, ^34^, ^42^, ^47^

Some mothers had already tried tactics to change their child's dietary behaviors (such as meals portion or fat content reduction).^29^, ^34^, ^35^, ^37^, ^46^, ^47^ Nevertheless, some mothers reported that in any attempt to change their children's dietary habits, they were challenged with various difficulties, including disobedience, anger, or crying from their children.^29^, ^34^, ^42^ Also, some parents thought that changing lifestyles entails neglecting their children,^34^ which should not be a family or group effort.^42^

#### 3.1.7 Sedentary lifestyles

Overall, children, parents, and teachers recognize sedentary behaviors as a risk factor for obesity.^36^, ^38^, ^45^, ^51^ For example, children's narratives constantly mention watching TV as their primary sedentary behavior. In addition, parents and teachers consider more sedentary behaviors (e.g., watching TV and/or using several electronic or indoor activities or lack of outdoor activities).^28^, ^34^, ^35^, ^36^, ^43^, ^47^

### 3.2 Kinship and social factors

#### 3.2.1 Family (immediate and extended)

Unanimously, children and adolescents acknowledge their mothers as primary care and primary food providers. Whether working or unemployed, mothers are recurrently pointed out as the primary provider and decision‐makers with the food offered at home.^33^, ^41^, ^45^, ^46^, ^48^, ^49^ According to children's descriptions of family dynamics, third parties (e.g., their father or grandparents) consent and indulge children's obesogenic behaviors, counteracting the mother's efforts to provide “healthier” foods.^34^, ^41^, ^42^, ^48^, ^51^

Mothers also take full responsibility for providing food to their children. Food decisions are frequently discussed and negotiated by children, who seem to have power in household food decisions. However, it depends on the availability and economic access of the mother or the family to carry them out.^33^, ^41^, ^46^ For working mothers or mothers with health issues (e.g., cancer), other female members from the extended family (e.g., daughters, aunts, or grandmothers) help with childcare and food provision.^33^, ^34^, ^38^, ^41^, ^42^, ^46^, ^47^, ^49^ Members of the extended families, especially aunts or grandmothers, seem to be more permissible and indulging regarding “junk” food given to children.^33^, ^34^, ^38^, ^41^, ^46^, ^47^, ^48^

#### 3.2.2 Child‐feeding practices

Children report that their feeding practices might be linked to their parent's schedule or work, and ready meals or takeaways are described as a simple solution for the lack of time of some working parents.^30^, ^32^, ^51^ Also, instead of taking homemade lunches, children report (and seemed enthusiastic about) getting money to buy food in/out of the school when there is “not enough time” to prepare something at home.^30^, ^33^, ^38^

Regardless of the socioeconomic status, giving money to children to buy some food was frequently reported by parents as an easy way to feed their children and give them some freedom to choose food.^30^, ^33^, ^34^, ^35^, ^36^

#### 3.2.3 Parental role

Some children report having different eating patterns in the presence/absence of their parents as if some foods were prohibited at home, and they try to consume them hidden.^32^, ^46^, ^48^, ^49^ All stakeholders considered mothers as the primary food providers and decision‐makers at home. Fathers are consistently reported as responsible for physical and recreational activities at home.^34^, ^41^, ^47^, ^48^, ^49^, ^51^ When fathers come into the narratives of children and mothers, these also revealed permissive feeding styles and behaviors (e.g., allowing children to eat “junk” food while watching TV).^29^, ^34^, ^37^, ^47^, ^49^, ^51^

Mothers complained that household responsibilities are not shared with their partners, making it challenging to feed their families adequately.^34^, ^47^, ^51^ In addition, for some mothers, accepting that their children have obesity generates thoughts of culpability or failure.^29^, ^42^ Moreover, mothers report having the need and responsibility to educate their children, including healthy eating.^29^, ^36^

#### 3.2.4 Friendship, social ties, and social support

Children highlight that their weight status and how peers react to it define friendships.^38^, ^39^ Some children reported that obesity represented a problem only when bullied at school or home.^32^, ^38^, ^39^, ^40^, ^45^ Some free codes at an individual level reveal the cruelty of how children with overweight and obesity can be treated and stigmatized by peers or even family members.^32^, ^38^, ^39^, ^40^, ^45^ Social isolation and stigma of children with overweight or obesity were clear in social^38^, ^39^, ^40^ and academic situations.^39^

#### 3.2.5 Responsibility of obesity

Across studies at an interpersonal and institutional level, it was frequently questioned who was responsible for children's weight status. Some parents “blamed” themselves for their children's weight status.^28^, ^29^, ^30^, ^32^, ^36^, ^37^, ^43^, ^51^ However, others justified themselves or gave answers that excluded them from their responsibility. Some parents attributed the full responsibility to their children.^32^, ^51^ Teachers and food industry interviewees unanimously believed childhood obesity is the responsibility of parents only.^30^, ^36^, ^50^ Moreover, teachers frequently reported facing challenges while trying to agree with parents regarding foods served in school.^28^, ^43^, ^51^

#### 3.2.6 Gender role

At an individual level, female adolescents perceive they need to take care of their weight for being women.^38^ Obesity seems to be distinguished only in females by other children or adolescents.^39^ Even female adolescents consider that “boys should be bigger than girls.”^39^

At an interpersonal level, mothers are gender‐biased towards childhood obesity. For example, mothers of children with obesity do not consider it a problem if their son(s) gain weight, whereas it is notorious and worrying if it is their daughter(s).^29^, ^42^

### 3.3 Economic factors

#### 3.3.1 Ability to purchase consumer goods

Depending on o their parents' occupations, some children might struggle to get more/less nutritious foods.^33^, ^41^ However, most parents (regardless of their socioeconomic status) provided money to their children, allowing them to purchase cheap “junk” food at schools or in school surroundings.^32^, ^33^, ^35^, ^38^, ^46^

#### 3.3.2 Setting (e.g., housing or school conditions)

Parents and teachers consider that not being able to do outdoor activities or having adequate open spaces is the major limitation for children to exercise. Insecurity in the streets and recreation areas, unsafe parks without lighting, lack of indoor spaces, poor infrastructure of public spaces, or lack of hygiene in public spaces were constantly mentioned by parents as a barrier for children using indoor or outdoor public facilities.^34^, ^35^, ^36^, ^43^, ^47^, ^51^ School teachers and staff also acknowledge the lack of suitable spaces or materials for physical or recreational activities outside and in school settings.^28^, ^36^, ^43^

#### 3.3.3 Employment type and stability

Children constantly mentioned that working mothers struggle to prepare homemade food, eat at home, or do physical activity.^30^, ^32^, ^33^, ^38^, ^41^, ^51^ Similarly, working duties were highlighted by parents as the crucial factor for not cooking^31^, ^34^, ^35^, ^36^, ^47^ or performing physical activities with their children.^36^, ^51^

#### 3.3.4 Cost of living

Some children, parents and teachers believe that nutritious food is expensive, hence justifying the lack of consumption/availability. Overall, children and parents believe that healthy lifestyles are linked to the capacity of being able to afford these.^31^, ^32^, ^34^, ^36^, ^41^ Children pointed out that money is indispensable for acquiring nutritional and “good quality” food and having a “better quality of life.”^33^, ^39^, ^41^, ^46^ Children and parents recognize that some physical activities (e.g., team sports like football) usually imply a cost, which is a major limitation for children's enrolment.^31^, ^34^, ^36^

### 3.4 Technological factors

#### 3.4.1 Access to computers/internet, social media, communication

Children, parents, and teachers recognize that children's primary sedentary behavior is linked to technological factors (e.g., easy and frequent access to TV).^28^, ^34^, ^35^, ^36^, ^38^, ^45^, ^47^, ^49^, ^51^ School teachers also acknowledge the access to technology as an easy way for children to swap the physical activities during school recess to using tablets or other devices.^28^, ^36^, ^43^

### 3.5 Political and legal factors

#### 3.5.1 Policy

Some teachers and school staff mentioned the lack of policies to regulate street food vendors near schools or norms and regulations for selling food and beverages within schools.^28^, ^30^, ^31^, ^38^, ^49^ Some school food vendors reported receiving a list of allowed/forbidden items with no rationale about the nutritional quality of products.^28^, ^50^ In one study, staff of infant public care centers reported that the organizational guidelines focused on hygiene, cost, and safety rather than the nutritional characteristics of food served to children.^43^ Some teachers also complain about the lack of strategies and guidelines to encourage physical activity among children during school hours.^43^, ^48^

### 3.6 Education factors

#### 3.6.1 Access to education

Parents and teachers highlighted that schools' health and nutrition education is limited to teaching the Mexican Eatwell plate (i.e., “Plato del Bien Comer”) and physical activity advice.^28^, ^51^ Some school staff felt not qualified or did not have the means to teach students healthy lifestyles.^28^, ^36^ Physical education at schools was considered by certain teachers as a “waste of time,” covering the minimum requirements established by the school system because of the lack of trained teachers, suitable spaces, or equipment.^51^

### 3.7 Biological factors

Children and some parents believe that having childhood obesity is a consequence of having older family members with obesity. Some children believe that obesity “happens at a certain age” (but not in childhood).^37^, ^40^, ^42^, ^45^, ^46^ Some parents believe that obesity occurs only in children genetically predisposed.^29^, ^37^ However, some parents recognize that despite a genetic predisposition to obesity, healthy habits can help prevent it.^42^

### 3.8 Quality assessment

Overall, the CASP tool showed that the results across studies were valid and relevant to this qualitative synthesis. All included studies had clear research objectives, and the qualitative methods were adequate and provided an explicit statement of the findings. Research designs were appropriate for all studies, except for one,^45^ in which the design was unclear. Most of the studies justified the settings and methods chosen for the studies. The recruitment strategy was appropriate for most of the studies, except for seven studies in which recruitment methods were unclear.^31^, ^32^, ^36^, ^38^, ^40^, ^45^, ^50^ Most studies failed to describe the relationship between the researchers and participants. Almost none of the studies critically assessed the role of the researchers, the potential bias during the research questions formulation or data collection. Six studies^28^, ^34^, ^36^, ^38^, ^45^, ^46^ did not report thoroughly how ethical standards were maintained during the study (Table 2).

**TABLE 2:** Methodological rigor and theoretical relevance of included studies

Study ID	Was there a clear statement of the aims of the research?	Is a qualitative methodology appropriate?	Was the research design appropriate to address the aims of the research?	Was the recruitment strategy appropriate to the aims of the research?	Was the data collected in a way that addressed the research issue?	Has the relationship between researcher and participants been adequately considered?	Have ethical issues been taken into consideration?	Was the data analysis sufficiently rigorous?	Is there a clear statement of findings?
Arroyo‐Lopez 2015^28^	✓	✓	✓	✓	✓	?	×	✓	✓
Avila‐Ortiz 2017^29^	✓	✓	✓	✓	✓	?	✓	✓	✓
Bonvecchio 2014^30^	✓	✓	✓	✓	?	?	✓	?	✓
Caballero‐Garcia 2017^31^	✓	✓	✓	?	?	?	✓	?	✓
Cabello‐Garza 2011^32^	✓	✓	✓	?	✓	?	✓	✓	✓
Cabello‐Garza 2014^33^	✓	✓	✓	✓	✓	?	✓	✓	✓
Cespedes 2012^34^	✓	✓	✓	✓	✓	?	×	✓	✓
Gallegos‐Martínez 2016^35^	✓	✓	✓	✓	✓	✓	✓	✓	✓
González‐Valencia 2018^36^	✓	✓	✓	?	?	?	×	✓	✓
Guendelman 2010^37^	✓	✓	✓	✓	✓	?	✓	✓	✓
Illescas‐Najera 2014^38^	✓	✓	✓	?	?	?	×	✓	✓
Levasseur 2017^39^	✓	✓	✓	✓	✓	✓	✓	✓	✓
Martínez‐Aguilar 2010^40^	✓	✓	✓	?	?	?	✓	✓	✓
Martinez‐Vargas 2022^41^	✓	✓	✓	✓	✓	✓	✓	✓	✓
Mendez 2014^42^	✓	✓	✓	✓	✓	✓	✓	✓	✓
Ortega‐Altamirano 2018^43^	✓	✓	✓	✓	✓	?	✓	✓	✓
Ortega‐Avila 2017^44^	✓	✓	✓	✓	✓	✓	✓	✓	✓
Pérez‐Gil Romo 2012^45^	✓	✓	?	?	?	✓	×	?	✓
Pérez‐Izquierdo 2020^46^	✓	✓	✓	✓	✓	?	?	✓	✓
Rodriguez‐Oliveros 2011^47^	✓	✓	✓	✓	✓	✓	✓	✓	✓
Théodore 2011^48^	✓	✓	✓	✓	✓	✓	✓	✓	✓
Théodore 2011^49^	✓	✓	✓	✓	✓	✓	✓	✓	✓
Théodore 2013^50^	✓	✓	✓	?	✓	?	✓	✓	✓
Turnbull 2019^51^	✓	✓	✓	✓	✓	✓	✓	✓	✓

*Note*: ✓ = yes; × = no; ? = unclear.

## 4 DISCUSSION

This systematic review evaluated qualitative evidence to examine cultural factors related to childhood and adolescent obesity in Mexico. Overall, 24 relevant studies were identified, including stakeholders' views, perceptions, or beliefs at different levels (i.e., individual, interpersonal, community or industry, and policy). Cultural values, beliefs, lifeways, kinship, and social factors strongly influence obesity‐promoting lifestyles among Mexican children and adolescents. Especially those related to food, food costumes and family (including the immediate and extended) were the descriptive themes most reported across participants as factors influencing children's obesity‐promoting lifestyles, weight, and health status.

Overall, family and food are central cultural values in Mexican culture. Different free codes identified at an individual or interpersonal level highlighted intergenerational influences across food and feeding practices among Mexican children or adolescents and their families. Family food environments are complex domains shaped by several factors, including family members' attitudes, knowledge, beliefs, and affordability or access to food.^52^ For Mexicans, eating with families (especially when eating out from home) is a part of the culture and is crucial for family bonding.^3^, ^51^ Still, childhood obesity‐promoting influences were also identified beyond food and family at all levels and across analytical themes (as shown in Figure 3). For instance, health and diet misconceptions, lack of social security, lack of resources to afford healthy lifestyles (e.g., accessing fresh food or team sports), and access to spaces where children can exercise safely were also identified.

Some cultural decisions, behaviors, individual experiences, perceptions, attitudes, or views identified in this review have also been found among Mexican families living in the United States.^3^, ^5^, ^6^, ^7^, ^53^ This suggests that some cultural factors related to obesity persist even after acculturation. For instance, it is widely agreed on the mother's role in their offspring's feeding practices and nutritional status.^3^, ^53^ The traditional maternal role of Mexican mothers (in Mexico and abroad) is to provide food and ensure a healthy family. However, narratives of Mexican mothers (regardless of the country they live in) show conflict, disappointment, and worry over not fulfilling such traditional roles for several reasons, including work duties, other household duties, or caring for more family members.^3^, ^53^ Similarly, mothers constantly criticize fathers for disrupting attempts to improve children's healthy lifestyles or for not sharing household responsibilities.^3^ Additionally, immediate and extended family members are relevant to children's feeding practices.^3^, ^53^ Interestingly, among Mexican families living in the United States, extended family members have been considered enablers for healthy change.^3^ In contrast, as shown in the current review, mothers in Mexico considered immediate and extended family members a fundamental barrier to adopting a healthier lifestyle.

Cultural expressions are dynamic and interactive with social, environmental, and biological conditions, either enhancing cultural predispositions or preventing cultural preferences towards childhood obesity.^5^ By using the Sunrise's Cultural and Social Structure Dimensions Enabler Model described within Leininger's theory,^20^, ^21^, ^22^ several cultural obesity‐related factors were identified at different levels in Mexico. How such factors interact is complex and not linear. As mentioned earlier in this section, food and family are core values of the Mexican culture. Food is a way of family bonding and a tradable currency that rewards or punishes children for certain behaviors, regulating the relationship between children and parents. In addition, limiting childhood obesity to an esthetic problem and health misconceptions (e.g., a “chubby child” is a healthy child) are two main issues that underpin and facilitate several other obesity‐promoting lifestyles. For example, the recurrent permissive parental (and extended family) feeding practices and the lack of children's interest or worry when weight matters are brought to the attention, focusing on food quantity rather than quality or serving unhealthy foods (e.g., sugary drinks) for acceptability among children. These factors are also worsened by the poor nutritional education reported at all levels (i.e., unclarity of what is “unhealthy” and their impact on weight and health outcomes), which aggravates other misconceptions and other obesity‐related factors. Moreover, food's predominant role in Mexican culture dismisses the significant role of physical activity or sedentary lifestyles in promoting or preventing obesity. In parallel, due to a nutritional and socioeconomic transition that has occurred in the last decades,^11^ socioeconomic (e.g., lower fresh food affordability and higher processed foods availability) or societal (e.g., unsafe or unsuitable outdoor spaces) factors also contribute to the complexity and interrelation of obesity‐related factors among Mexican children and adolescents.

The current review found a conflicting narrative between Mexican parents, teachers, school staff, and industry representatives about childhood and adolescent obesity “responsibilities.” Parents were usually the ones to “blame” for childhood obesity. Nevertheless, stakeholders (at all levels) need to acknowledge that childhood and adolescent obesity is a shared responsibility that requires action at several levels to achieve meaningful healthy lifestyles and effective health improvements.^24^, ^25^, ^54^

Recently, different nationwide strategies to tackle obesity among the general population have been implemented in Mexico. For instance, a 1 peso/liter tax on sugar‐sweetened beverages^55^, ^56^ and a front‐of‐pack labeling system have been implemented.^57^ Nonetheless, interpersonal, community, institutional, or industry quotes (especially those from schoolteachers and staff) show a lack of a sense of priority by institutions, policymakers, or the government for obesity prevention when considering childhood obesity treatment or prevention. In this sense, schools are essential for childhood obesity prevention and adequate communication among relevant stakeholders. Previous evidence has shown that healthy eating and physical activity can be promoted within schools.^54^, ^58^, ^59^ Schools are a suitable environment where relevant stakeholders (e.g., parents, teachers, and other stakeholders) can work closely towards the same goal.^54^

Some limitations of the current review include that only five studies^28^, ^39^, ^40^, ^44^, ^46^ consider the adolescent population (between 13 and 18 years). Adolescents get much more autonomy in their lifestyles than children. Hence, results might not reflect the overall perceptions or beliefs of adolescents. Also, understanding the continued trajectory of obesity in young people and the level of involvement might vary according to age. Like other reviews,^3^, ^53^ most of the codes retrieved were from female participants (e.g., mothers or teachers). This might cause a sex bias, with more female than male perspectives, particularly at an interpersonal level (i.e., parents and teachers). Finding a considerably higher number of codes from female participants is perhaps a reflection of Mexican society, with females being the principal caregivers of children at schools and homes. As shown in Figure 2, the retrieved evidence comes from 16 out of 32 states, not displaying a nationwide picture. Additionally, not all the studies provided detailed sociodemographic characteristics of the participants, challenging the interpretation of results in the light of the different socioeconomic and demographic backgrounds, which might affect participants' views.

To our knowledge, this is the first review of cultural factors related to obesity among children and adolescents within the Mexican culture context (not as an ethnic minority). An exhaustive search was performed across 10 databases and one search engine in two languages, which helped us to capture relevant publications. As part of the COMO project, a search for grey literature relevant to childhood obesity in Mexico,^15^ but no relevant qualitative reports were found. Also, by guiding this review through the CCT, we seek to provide health professionals and researchers with a culturally congruent care guide to understand the culture‐specific needs to be considered in future efforts to prevent or treat childhood obesity in Mexico.

Without considering cultural factors, decisions, behaviors, individual experiences, perceptions, attitudes, or views inherent to the target population, any analysis of socioeconomic influences, behaviors, or environmental causes of obesity is incomplete. This review identified cultural factors related to obesity among children and adolescents in Mexico, showing future research or care service development areas. Culturally relevant interventions and practical adaptation strategies are vital to improving health promotion efforts to tackle obesity. When designing interventions, it is crucial to consider the target population's unique cultural values, beliefs, socioeconomic status, and environment.^60^

Intervention designers and policymakers should incorporate as many cultural elements as possible within obesity prevention or treatment interventions and policies among Mexican children and adolescents. Building a framework to culturally adapt interventions (or specific elements of the intervention) would be helpful to guarantee that future efforts are more appealing to participants. Also, efforts to comprehend and incorporate the core cultural values into health interventions can be enhanced by the involvement of both experts and participants in the design of interventions and engaging with the community and other stakeholders.^61^, ^62^ Moreover, the result of the current review highlights the need to move beyond traditional nutrition education techniques that intend to produce individual behavioral changes to culturally tailored approaches that involve stakeholders at different levels, ensuring that obesity prevention and treatment is a shared responsibility. Additionally, efforts need to be coordinated and integrated from national‐level policies to community‐level programs, influencing specific activities in communities, schools, and homes to promote healthier lifestyles in families, thus in children and adolescents.

## CONFLICT OF INTEREST

MA‐M, LL‐C, NLGF, MG‐B, and CFMG have no conflict of interest to declare. YYGG received funding from Abbott to write two book chapters in 2020 and Bonafont to present in a congress in 2016.

## Supporting information


**Table S1.** Search strategy
**Table S2.** Free Codes Categorisation Guideline for Reviewers

## Appendix A.

**TABLE A1:** Main characteristics of included studies

Study ID city, state	Aim	Setting	Participants	Qualitative methods
Arroyo‐Lopez 2015^28^ Toluca, Mexico State	To analyze the implementation and effectiveness of the actions carried out, from the perspective of the responsible school agents, to establish modifications to improve its effectiveness	Public secondary schools (serving adolescents from 12–15 years old)	Twelve participants, including ten school staff (i.e., two school principals, assistant principals, five teachers, and three physical education teachers), one food vendor, and one nutrition advisor from one of the secondary schools. All implemented, supervised, or evaluated preventive actions defined by the government to control overweight and obesity in schools	Type of study: Qualitative study Framework or theory used: cognitive social theory, including participatory action research and case study Data collection: 10 and 12 open‐ended questions were designed based on the profile of each critical informant. Duration of ~1 h Analysis: Thematic analysis Date of the study: NR
Avila‐Ortiz 2017^29^ Monterrey, Nuevo Leon	To explore and describe mothers' perceptions concerning the body weight of their children	Recruitment at a public primary school in an urban area, interviews were administered either in homes or schools	Ninety‐one middle‐class mothers (23–51 years) with children between 7 and 11 years. 8% of the children had underweight, 38% normal weight, 15% overweight, and 38% obesity. The majority were married, worked as homemakers and possessed an education level that was a predominately secondary school (26%) or college degrees (24%)	Type of study: Qualitative study Framework or theory used: None reported. Collection: Semi‐structured interviews based on a guide that contained subjects Analysis: Thematic analysis Date of the study: June 2011 and December 2013
Bonvecchio 2014^30^ Mexico City	To describe the methods and key findings of formative research conducted to design a school‐based program for obesity prevention	Public primary schools serving low SES areas and receiving the national school breakfast program. Schools had at least one schoolyard facility for PA, over 300 students, and two or more classrooms per grade	~70 participants, including ~50 students from 9 to 11 years of age in five focus groups with 6–10 children each, four parents, four schoolteachers, four food vendors, four school principals	Type of study: Formative research was conducted to design a school‐based program that included qualitative input Framework or theory used: Simplified ecological model based on McLeroy's schema. The information was analyzed using the grounded theory and the phenomenology theory Collection: Discussion groups were conducted with children separated by sex. The interviews were conducted with parents, school staff, and food vendors, following a semi‐structured guide to direct the conversation Analysis: All in‐depth interviews and discussion groups were taped and transcribed. Date of the study: NR
Caballero‐Garcia 2017^31^ Chilpancingo (Guerrero), Puerto Vallarta (Jalisco), Coatetelco (Morelos) and Hermosillo (Sonora)	To evaluate the actions of an educational intervention in food, oral health, and hygiene developed within the framework of the healthy schools' program in four states of the Mexican Republic	Four elementary public schools in municipalities of medium to high marginalization within the four taking part states	Twenty‐nine teachers (six in Morelos, ten in Guerrero, seven in Jalisco and six in Sonora)	Type of study: Mixed‐methods study. Framework or theory used: Taylor and Bogdan's method Collection: Focus group interviews. Analysis: The analysis was carried out through the following phases: (a) identification of topics and development of concepts and propositions, (b) data coding according to the large blocks of identified topics, and (c) analysis according to the context and the people interviewed Date of the study: 2006–2008
Cabello‐Garza 2011^32^ Monterrey, Nuevo Leon	To describe the beliefs, conceptions, and perceptions that mothers of children with obesity have about their children's body image and eating practices and the factors that motivate, facilitate, or are obstacles to a healthier lifestyle	Urban public elementary schools of middle, lower‐middle, and low SES	Eight mothers of children with obesity who were in the 4th and 5th grade (9–11 years of age)	Type of study: Qualitative Framework or theory used: Taylor and Bogdan's method Collection: Semi‐structured interviews Analysis: The analysis was carried out through the following phases: (a) identification of topics and development of concepts, (b) data coding according to the large blocks of identified topics, and (c) analysis according to the context and the people interviewed Date of the study: 2008–2009
Cabello‐Garza 2014^33^ Monterrey, Nuevo Leon	To identify intrapersonal, family and school contexts related to feeding practices, weight gain, and some problems that make it challenging to have a healthy lifestyle for children with obesity	Elementary schools in urban areas	Twelve children enrolled in 4th and 5th grade (9–11 years of age) that had obesity	Type of study: Inductive‐qualitative study Framework or theory used: Ecologic Theory from Bronfenbrenner and triangulation of topics techniques Collection: Semi‐structured qualitative interview based on the visualization of children's figures that showed a different contour to elicit identification and free speech of each of the children Analysis: Content analysis with the speeches, a series of topics and ideas were structured, from which categories emerged that were systematized and ordered, supported by a software Date of the study: 2011
Cespedes 2012^34^ Mexico City	To examine caregivers' perceptions of the role of primary care in childhood obesity management, understand the barriers and facilitators of behavior change, and identify opportunities to strengthen obesity prevention and treatment in clinical settings	Primary care clinics, From one Ministry of Health Clinic (public, low SES) and 4 Social Security Institute Clinics (insured, higher SES). Interviews took place in the clinics or in the participant's home	Fifty‐two parents and caregivers of children (2–5 years old) with overweight or obesity receiving preventive or curative health care for problems unrelated to weight with diverse SES (32 affiliated with the Social Security Institute and 20 with the Ministry of Health care system). 83% were mothers; the rest were aunts, grandmothers, and fathers. The mean age was 33 years. Education levels varied among participants and recruitment institutions. Also, 45% of those interviewed in the Ministry of Health care system reported their occupation as homemakers, compared with 60% at the Social Security Institute care system	Type of study: Qualitative study Framework or theory used: NR Collection: Trained research staff conducted in‐depth interviews using a structured discussion guide. Each interview lasted ~40 min Analysis: Systematic thematic analysis. Using a random sample of 15 interviews, three researchers developed a thematic catalog of codes Date of the study: July 2010–June 2011
Gallegos‐Martínez 2016^35^ San Luis Potosi City, San Luis Potosí	To determine the social representations attributed by caregivers, teachers, and children to feeding, health, and nutrition and the school breakfast program for children	Four preschools and school educational facilities assigned to the school breakfast program for the family's comprehensive development program	Seventy‐five participants, including 33 mothers, three grandmothers, one father, 30 children from 3 to 7 years of age, and eight teachers (one to three respondents per facility). Parent's education included: 37.8% had incomplete or complete preparatory (37.8%). In addition, 38.7% of mothers' occupations were full‐time household work, including the father. The teaching staff ages ranged from 28 to 49 years of age and over 5 years of teaching experience	Type of study: Clinical‐qualitative health approach Framework or theory used: Based on social representations following Moscovici's approach Collection: Semi‐structured interviews for adults, group interviews for children telling them they were “playing as reporters” and being interviewed. Analysis: Content analysis modality thematic analysis. The interviews lasted ~30 min Date of the study: June–July 2014
González‐Valencia 2018^36^ Hermosillo, Sonora	To implement a participatory reflection to identify mediating variables of behavior and environment that will lead to a program for preventing obesity in childhood with a public health approach	17 public schools (4 preschools and 13 elementary schools that benefited from the school breakfast program)	Five hundred ten participants, including 186 parents, 167 teachers, 130 school children, and 48 preschool children. No characteristics of participants were provided	Type of study: Qualitative Framework or theory used: PRECEDE‐PROCEED (predisposing, reinforcing, and enabling constructs in educational diagnosis and evaluation‐policy, regulatory, and organizational constructs in educational and environmental development) model Collection: Focus groups, 10 participants per group, 17 focus groups with parent parents, 17 teachers, 13 school children, and four con preschool children, followed a thematic guide on healthy eating and physical activity Analysis: Thematic analysis. Focus groups were recorded and transcribed. Themes and subthemes were analyzed with the help of software Date of the study: January–December 2014
Guendelman 2010^37^ Jalisco and Guanajuato	To assess maternal perceptions of their child's actual and ideal body size; what it means to be overweight during infancy and childhood; and factors contributing to childhood overweight among low‐income women	Focus group occurred in clinics, community centers, or schools	Thirty‐two mothers who had children 4–6 years old. Mean age 30.7 (5.7); Education means years 5.4 (3.1) and 42.3% living in rural areas which benefited from “Oportunidades” (a conditional cash transfer program that provides money and food supplements for low‐income children)	Type of study: Mixed methods study Framework or theory used: NR Collection: Focus‐group discussions were conducted with a purposive sample by an experienced social worker. Focus‐group discussions were audio‐recorded, transcribed, and followed by a debriefing Analysis: Thematic analysis Date of the study: March 2006 and January 2008
Illescas‐Najera 2014^38^ Veracruz City, Veracruz	To identify the eating behavior of school‐aged children with obesity and to identify food preferences	School setting. No further details were provided about the setting	Fourteen children (11–12 years) with obesity. Eight were male and six females	Type of study: Qualitative study Framework or theory used: Methods suggested by Strauss & Corbin Collection: Face‐to‐face interviews for data collection and observation guide to inquire about their preferences in selecting food in and out of schools. Lasted ~30 min Analysis: Thematic analysis Date of the study: NR
Levasseur 2017^39^ Mexico City	To enrich the conceptual foundations of the causal relationship between childhood overweight and obesity and an individual's ability to study	Primary and secondary schools in different areas included a private school with nine participants (upper‐middle SES) and a public school with sixteen participants (very low SES)	Twenty‐nine adolescents (11–15 years old. Fifteen were male and 14 female	Type of study: Qualitative study Overall methods: grounded theory Framework or theory used: NR Collection: In‐depth interview techniques, ~40 min Analysis: Thematic analysis Date of the study: June and August 2016
Martínez‐Aguilar 2010^40^ Tamaulipas City, Tamaulipas	To explore perceptions about obesity among students at a public school	Public secondary school	Twenty‐four adolescents with obesity in between 11 and 15 years old. Fourteen were female and ten males	Type of study: Qualitative Framework or Theory used: NR Collection: a semi‐structured interview that took ~30 min applied until data saturation and until the meaning was understood. Analysis: Thematic analysis Date of the study: March and June 2008
Martínez‐Vargas 2022^41^ City NR, Morelos	To identify determinants of unhealthy eating by examining the perspectives and experiences of low‐income Mexican women with a child at home.	Kindergartens located in marginalized areas	Thirty women (mean age 30.5 years) who had at least one child enrolled in one of the four participating kindergartens were eligible. In addition, 90% were married, and 63.3% had completed secondary school.	Type of study: Qualitative Framework or theory used: Dahlgren and Whitehead's social determinants of health model. Collection: Trained research assistants carried out semi‐structured interviews and focus groups (6–8 participants each). Interviews lasted ~60 min, and focus groups ~45 min. Also, individual, in‐depth interviews Analysis: All data were transcribed verbatim and analyzed with the help of qualitative analysis software and through a thematic analysis Date of the study: May–June 2018
Mendez 2014^42^ City NR, Yucatan	To understand caregivers' perceived role in addressing their children's obesity within the family context and identifying topics that could be considered when providing health care in similar socio‐cultural environments	Local cardiometabolic unit of a clinic	Forty‐eight parents or principal caregivers (including mothers, fathers, grandmothers, an aunt, and a sister) of urban children (6–13 years) with a confirmed diagnosis of morbid obesity and under treatment at the cardiometabolic unit. The average age of 34 years. 39.8% had a high school degree, and 8.3% had a bachelor's degree	Type of study: Qualitative Framework or theory used: Constant comparative method is a selective technique drawn from grounded theory Collection: Open‐ended focus group interviews and group discussions where participants share and discuss their perceptions. Analysis: agreement and disagreement points among participants Date of the study: April and July 2011
Ortega‐Altamirano 2018^43^ Mexico City	To identify strengths, weaknesses, opportunities, and threats perceived by childcare staff for preventing childhood overweight	Six public childcare centers in four different regions of the city	Eighty‐nine staff members from six public childcare centers included 39 caregivers and 32 kitchen staff, and 18 semi‐structured in‐depth interviews with six directors, six teachers, and six dietitians. Age range was 20–62 years old. The educational level of the caregivers and kitchen staff was 6–9 years, whereas the directors, teachers, and dietitians had 12 or more years of education. Half of the participants had over 10 years of labor seniority. 81% of the participants in the study were women	Type of study: Qualitative study Framework or theory used: an interpretative phenomenological approach Collection: 18 in‐depth, semi‐structured interviews and 12 focus groups (6 participants per group). The interviews lasted 50 min, and the focus groups lasted 93 min on average. Transcripts by comparing the audio recording with the texts and their field notes Analysis: Thematic analysis was conducted with an analytical process of a circular structure of understanding. Also, SWOT analysis by categorizing perceptions was used Date of the study: October 2010–February 2012
Ortega‐Avila 2017^44^ Hermosillo, Sonora	To explore awareness and perceptions of the sugar‐sweetened beverage tax implemented in Mexico in 2014 among a sample of Mexican adolescents	Participants were approached and recruited through participation in an earlier cross‐sectional online survey. However, interviews were carried out in public spaces or in participants' homes	Twenty‐nine adolescents, 16 females, and 13 males, mean age 17.0 (SD 1·4) years). In addition, 55% of participants had normal weight, 25% of the men and 10% of the women had overweight	Type of study: Qualitative Overall methods: Semi‐structured interviews lasting an average of 35 min (27–50 min) Framework or theory used: The framework approach Collection: Face‐to‐face individual interviews. Analysis: Thematic analysis of the interview transcripts Date of the study: April–May 2016
Pérez‐Gil Romo 2012^45^ Cacalotepec, Villa de Tututepec, Oaxaca	To explore the perception of obesity and nutrition in children from rural areas	School rural public setting, in a community with primary services shortage	Fifty‐eight afro mestizos and indigenous children from 9–12 years old. 28 were males and 30 females	Type of study: Qualitative Framework or theory used: NR Collection: Interviews with a playful mode to engage with children. Closed‐ended questions and interviews Analysis: Only those statements related to the perceptions of obesity were considered in the analysis Date of the study: March 2012
Pérez‐Izquierdo 2020^46^ Abala, Yucatan,	To identify types of modern industrialized foods that adolescents with overweight and obesity consume and their perception of them	High schools from rural e indigenous areas	~50 adolescent students from four high schools from rural e indigenous (Maya) backgrounds	Type of study: Mixed methods study Framework or theory used: “Theory saturation.” Collection: Five focus groups composed of 8–12 students with overweight or obesity Analysis: Thematic analysis Date of the study: NR
Rodriguez‐Oliveros 2011^47^ Mexico City	To explore perceptions or practices of key obesity‐related issues among parents of preschool‐aged children attending childcare centers	Five childcare centers of the Social Security Institute from different SES areas	Thirty‐eight parents of preschool children and 29 mothers and nine fathers from 20 to 63 years	Type of study: Qualitative Framework or Theory used: NR Collection: Focus groups following an interview guide to explore the specific topics. Each group included 6–11 participants. The mean duration of the focus group sessions was 73 min. Analysis: Content analysis by focusing on lexical, expression, and relations text analysis Date of the study: conducted in 2010
Théodore 2011b^48^ Mexico City	To identify the main social representations related to school meals present in the discourse of different actors within the schools and their relationship with the obesogenic school environment	Ten elementary public schools with standard population and organizational criteria (morning shift, low SES, the beneficiary of the Federal School Breakfast program, with a population greater than 300 students, and at least two classrooms per grade)	An unclear number of participants. School principals, physical education teachers, a teacher per group responsible for running the school cooperative, mothers, food suppliers, and 4th‐ and 5th‐grade children were included	Type of study: Qualitative Framework or theory used: Phenomenology Social Theory as a central concept of social representation Collection: Four in‐depth interviews were conducted for each adult school actor and ten discussion groups with children (five regarding food and five regarding physical activity). Analysis: Continuous analytic procedure of comparison among the discourses Date of the study: February and June 2006
Théodore 2011^49^ Mexico City	To analyze the importance of the cultural factors that today motivate Mexican children to consume sugary drinks and examine their implications for designing programs to promote healthy lifestyles	Four elementary public shared standard population and organizational criteria (morning shift, low SES, the beneficiary of the Federal School Breakfast program, with a population greater than 300 students, and at least two classrooms per grade)	Fifty‐three children (9–10 years) 25 were female, and 28 were males 9–10 years	Type of study: Qualitative Framework or theory used: Phenomenology social theory Collection: Interviews with two participants at a time and discussion groups Analysis: recorded and subsequently transcribed were analyzed by applying a continuous analytic procedure of comparison among the discourses Date of the study: September 2008 and November 2009
Théodore 2013^50^ Mexico City	To identify barriers and opportunities for regulating food and beverage advertising to children.	Unclear	Fourteen key informants from the congress, private sector, officials from the ministry of health and academics	Type of study: Qualitative study Framework or theory used: NR Collection: Semi‐structured interviews Analysis: Thematic discourse analysis. Date of the study: 2011
Turnbull 2019^51^ Mexico City	To understand childhood obesity in the Mexican context	Three elementary schools in a low SES area in Mexico City	Sixty children (8–12 years old), 24 parents (mainly mothers 25–49 years old), and 28 teachers (22 classroom teachers and six physical education teachers). 47% of the children were males, and 52% were females. 51% of the children had a normal BMI, 24% had overweight, and 23.4% had obesity. The majority (66%) of parents had completed junior high school, 52% were employed, and only 52% held formal jobs. Almost all homes (98%) in the sample had electricity, running water, and sewage; however, 13% did not have bathroom facilities within their home	Type of study: Qualitative Framework or theory used: Socio‐ecological model Collection: Semi‐structured interviews Analysis: Thematic discourse analysis Date of the study: NR

Abbreviations: BMI, body mass index; min, minutes; SES, socioeconomic status; SWOT, strengths, weaknesses, opportunities, and threats approach.

## Appendix B.

### COMPLEMENTARY LIST OF RESULTS INCLUDING THOSE WITH A LOWER DENSITY AND ONLY PRESENTED IN ONE STUDY

#### Cultural values, beliefs, lifeways

*Food and food customs*

All relevant codes are reported in the main manuscript.

*Illness and death*

All relevant codes are reported in the main manuscript.

*Dietary habits*

All relevant codes are reported in the main manuscript.

### Physical activity

Overall, children consider physical activity a weight management strategy rather than a recreational activity or healthy lifestyle.^2,7,12,19,22^ Children perceive that those peers with obesity might experience some difficulties when exercising because of the excess weight.^7,19,22^ Parents also consider physical activity a weight management strategy,^10,13,15,24^ and some mentioned challenges while trying to perform physical activities with their children (e.g., children's dislike, costs, safety, or lack of time).^10,11,13,15,17,24^

Teachers and health professionals at schools acknowledge the importance of physical activity in obesity prevention.^11,12,18^ Nevertheless, most report not having a structured physical education class or lacking knowledge on motivating children to engage effectively in physical activities.^11,12,18^ Some teachers also mentioned they did not encourage physical activities since children are “at risk of getting injuries” or “generating complaints from parents.”^11^

*Eating rituals*

All relevant codes are reported in the main manuscript.

*Beliefs about health*

All relevant codes are reported in the main manuscript.

*Sedentary lifestyles*

All relevant codes are reported in the main manuscript.

#### Kinship and social factors

*Family (immediate and extended)*

All relevant codes are reported in the main manuscript.

*Child‐feeding practices*

All relevant codes are reported in the main manuscript.

*Parental role*

All relevant codes are reported in the main manuscript.

*Friendship, social ties, and social support*

Children highlight that their weight status and how peers react to it define friendships.^2,19^ Some children reported that obesity represented a problem only when bullied at school or home.^2,7,16,19,22^ Some free codes at an individual level reveal the cruelty of how children with overweight and obesity can be treated and stigmatized by peers or even family members.^2,7,16,19,22^ Some children experience stress from feeling rejected by their peers.^19,22^ Social isolation and stigma of children with overweight or obesity were evident in social^2,19,22^ and academic situations.^19^ Some parents showed concerns about how peers might react to their child's excess weight.^24^ One study reported parents witnessing their children's abuse from peers or another family members^10^.

*Responsibility of obesity*

All relevant codes are reported in the main manuscript.

*Gender role*

All relevant codes are reported in the main manuscript.

#### Economic factors

*Ability to purchase consumer goods*

All relevant codes are reported in the main manuscript.

*Setting (e.g., housing or school conditions)*

Parents and teachers consider that being outside or in open spaces is the major limitation for children to exercise. Insecurity in the streets and recreation areas, unsafe parks without lighting, lack of indoor spaces, poor infrastructure of public spaces, or lack of hygiene in public spaces were constantly stated by parents as a barrier for children using outdoor public facilities.^4,11–13,15,17^ School teachers and staff also acknowledge the lack of suitable spaces or materials for performing physical or recreational activities outside and in school settings.^11,12,18^ In addition, the lack of safe water was highlighted by one study as a primary barrier to consuming plain water at schools.^5^

*Employment type and stability*

Children constantly mentioned that working mothers struggle to prepare homemade food, eat at home, or do physical activity.^1,2,8,9,16,17^ Similarly, working duties were highlighted by parents as the crucial factor for not cooking^4,12,13,15,21^ or performing physical activities with their children.^12,17^ Teachers also declare that the lack of time for working parents is a critical factor for feeding children adequately or engaging in physical activity.^11^

*Cost of living*

Some children, parents, and teachers know that some healthy behaviors and nutritious foods are linked to the capacity to pay for them.^9,12,13,16,21^ Children pointed out that money is indispensable for acquiring nutritional and “good quality” food and for having a “better quality of life.”^1,3,9,19^ Children's free codes show they associate nutritious food with high prices, justifying the lack of consumption/availability.^21^ Children and parents recognize that some physical activities (e.g., team sports like football) usually imply a cost, a significant limitation for enrollment.^12,13,21^

*Socioeconomic status*

Some free codes showed that children living in rural marginalized areas (regardless of the city or town) or with low socioeconomic status might experience more difficulties accessing healthy food or safe spaces to perform recreational facilities.^3,8–10,17^

*Transportation*

In large cities (e.g., the capital, Mexico City), long commuting times and transportation issues were highlighted by interviewees as a primary factor of lack of time to cook healthy food or exercise with their children.^11,15,17^

*Food insecurity*

School teachers were the only stakeholders to perceive food insecurity among children.^4,11,21^ They described knowing which children came from financially struggling families and reported that some children either go to school with no breakfast, eat “cheap food,” insufficient, or very‐inferior quality food.^4,11,21^

*Health care access, health care quality*

Parents in one study emphasized the relevance of health care in their children's obesity diagnosis or treatment.^13^ However, as most parents do not perceive childhood obesity as a health issue, they find out about the weight‐related matters once they take their children to other medical procedures (e.g., vaccination).^13^

#### Technological factors

*Access to computers/internet, social media, communication*

All relevant codes are reported in the main manuscript.

#### Political and legal factors

*Policy*

All relevant codes are reported in the main manuscript.

*Government*

Only one study^20^ interviewed academics, deputies, and policymakers, which pointed out the importance (and the lack) of national regulations and governmental actions to protect children's right to healthy eating, necessary for Mexican children and adolescents' growth and proper development.

#### Education factors

*Access to education*

All relevant codes are reported in the main manuscript.

*Education disparity*

Only one study reported the effects that obesity might have on learning outcomes. The authors concluded that adolescents' stigmatization and social isolation with obesity generate a substantial education disparity. Adolescents with obesity were at a higher risk of missing classes, not working teams, or not returning homework^19^.

*Child labor*

Only one study^21^ stated that children from rural or marginalized areas are essential for the daily subsistence of the household, which requires the work of both parents and, sometimes, of the children making it challenging for families, especially children, to maintain healthy lifestyles.

#### Biological factors

All relevant codes are reported in the main manuscript.

#### References from this appendix

1. Cabello Garza ML, Vázquez‐González S. Prácticas alimentarias y obesidad infantil. Cultura regional y factores intrapersonales familiares y escolares. 2014;

2. Illescas Nájera I, Acosta Cervantes MdC, Sánchez Rovelo MC, Del Socorro Mateu Armand MV, Garcimarrero Espino E. Estudio de la conducta alimentaria de escolares obesos de la ciudad de Xalapa, Veracruz (México) mediante entrevista personalizada. *Nutr clín diet Hosp*. 2014;34(2):97‐102.

3. Pérez‐Izquierdo O, Cárdenas‐García S, Aranda‐González I, Perera‐Ríos J, Castillo MdRB. Consumo frecuente de alimentos industrializados y su percepción en adolescentes indígenas Mayas con sobrepeso y obesidad. *Ciência & Saúde Coletiva*. 2020;25:4423‐4438.

4. Gallegos‐Martínez J, Reyes‐Hernández J. Representations by Caregivers, Teachers, and Children on Food, Nutrition, Health, and School Breakfast Contributions for the" ESNUT" Nutritional Stabilization Program. *Investigacion y educacion en enfermeria*. 2016;34(2):368‐377.

5. Théodore F, Bonvecchio A, Blanco I, Irizarry L, Nava A, Carriedo A. Significados culturalmente construidos para el consumo de bebidas azucaradas entre escolares de la Ciudad de México. *Revista Panamericana de Salud Pública*. 2011;30:327‐334.

6. Théodore FL, Bonvecchio Arenas A, Blanco García I, Carreto Rivera Y. Representaciones sociales relacionadas con la alimentación escolar: el caso de las escuelas públicas de la Ciudad de México. *Salud colectiva*. 2011;7:215‐229.

7. Romo SEP‐G, Esparza JIM, Juárez AGR. Obesidad y alimentación: percepción de un grupo de niños y niñas de la costa de Oaxaca. *Infancias Imágenes*. 2012;11(2):16‐26.8. Bonvecchio A, Théodore FL, Safdie M, et al. Contribution of formative research to design an environmental program for obesity prevention in schools in Mexico City. *salud pública de méxico*. 2014;56(s2):139‐147.

9. Martínez‐Vargas L, Vermandere H, Bautista‐Arredondo S, Colchero MA. The role of social determinants on unhealthy eating habits in an urban area in Mexico: A qualitative study in low‐income mothers with a young child at home. *Appetite*. 2022/02/01/ 2022;169:105852. doi:https://doi.org/10.1016/j.appet.2021.105852

10. Mendez N, Barrera‐Pérez M, Palma‐Solís M, Dickinson F, Uicab‐Pool G, Castillo‐Burguete M. You are not fat, you are hermosa»: Mexican caregivers share their perceptions about their role supporting their morbidly obese children. *Hisp Health Care Int*. 2014;12(4):174‐82.

11. Ortega‐Altamirano DV, Rodríguez‐Oliveros G, González‐Unzaga MA, Reyes‐Morales H. Perceptions of childcare staff for preventing overweight in Mexican preschool children: A SWOT analysis. *salud pública de méxico*. 2018;60:166‐174.

12. González Valencia DG, Grijalva Haro MI, Montiel Carbajal M, Ortega Vélez MI. Identificación de factores predisponentes, reforzadores y capacitadores para una alimentación y actividad física adecuadas en escolares sonorenses. *Región y sociedad*. 2018;30(72)

13. Cespedes E, Andrade GOM, Rodríguez‐Oliveros G, et al. Opportunities to strengthen childhood obesity prevention in two mexican health care settings. *International journal of person centered medicine*. 2012;2(3):496.

14. Guendelman S, Fernald LC, Neufeld LM, Fuentes‐Afflick E. Maternal perceptions of early childhood ideal body weight differ among Mexican‐origin mothers residing in Mexico compared to California. *Journal of the american dietetic association*. 2010;110(2):222‐229.

15. Rodríguez‐Oliveros G, Haines J, Ortega‐Altamirano D, et al. Obesity determinants in Mexican preschool children: parental perceptions and practices related to feeding and physical activity. *Archives of medical research*. 2011;42(6):532‐539.

16. Garza MLC, Reyes DDJ. Percepción de las madres de niños con obesidad sobre los hábitos alimenticios y sus responsabilidades en la alimentación de los hijos. *Revista salud pública y nutrición*. 2011;12(1)

17. Turnbull B, Gordon SF, Martínez‐Andrade GO, González‐Unzaga M. Childhood obesity in Mexico: A critical analysis of the environmental factors, behaviours and discourses contributing to the epidemic. *Health psychology open*. 2019;6(1):2055102919849406.

18. Arroyo‐López PE, Carrete‐Lucero L. Alcance de las acciones para prevenir el sobrepeso y la obesidad en adolescentes. El caso de las escuelas públicas mexicanas. *Revista Gerencia y Políticas de Salud*. 2015;14(28):142‐160.

19. Levasseur P, Ortiz‐Hernandez L. Comment l’obésité infantile affecte‐t‐elle la réussite scolaire? Contributions d’une analyse qualitative mise en place à Mexico. *Autrepart*. 2017;(3):51‐72.

20. Théodore F, Juárez‐Ramírez C, Cahuana‐Hurtado L, Blanco I, Tolentino‐Mayo L, Bonvecchio A. Barreras y oportunidades para la regulación de la publicidad de alimentos y bebidas dirigida a niños en México. *salud pública de méxico*. 2014;56:s123‐s129.

21. Caballero‐García CR, Flores‐Alatorre JF, Bonilla‐Fernández P, Arenas‐Monreal L. Experiencias de promoción de la salud en escuelas de nivel primario en México. *Mem Inst Invest Cienc Salud (Impr)*. 2017:22‐32.

22. Martínez‐Aguilar M, Flores‐Peña Y, Rizo‐Baeza M, Aguilar‐Hernández RM, Vázquez‐Galindo L, Gutiérres‐Sánchez G. 7th to 9th grade obese adolescents’ perceptions about obesity in tamaulipas, Mexico. *Revista Latino‐Americana de Enfermagem*. 2010;18:48‐53.

23. Ortega‐Avila AG, Papadaki A, Jago R. Exploring perceptions of the Mexican sugar‐sweetened beverage tax among adolescents in north‐west Mexico: a qualitative study. *Public health nutrition*. 2018;21(3):618‐626.

24. Ávila‐Ortiz MN, Castro‐Sánchez AE, Zambrano‐Moreno A. Mexican mothers' perceptions of their child's body weight. *Health & social care in the community*. 2017;25(2):569‐577.
