# Supplementary material for: Cultural factors related to childhood and adolescent obesity in Mexico: A systematic review of qualitative studies
Source: Obes Rev. 2022 May 19;23(9):e13461. doi: 10.1111/obr.13461 (PMC9541705; doi:10.1111/obr.13461)
Supplement: Supplementary file 1 — Table S1. Search strategy Table S2. Free Codes Categorisation Guideline for Reviewers [file OBR-23-e13461-s001.docx]

Supplementary table 1: List of participating centres

| CENTRE | num cases | % |
| --- | --- | --- |
| Hospital Universitario Virgen Arrixaca, Murcia. Spain | 82 | 26,9 |
| Favaloro Foundation University Hospital, Buenos Aires. Argentina | 41 | 13,4 |
| University of Michigan Hospital, Ann Arbor, MI, USA | 29 | 9,5 |
| Hospital of the University of Pennsylvania, Philadelphia, Pennsylvania, USA | 26 | 8,5 |
| Hospital General Universitario Gregorio Marañón, Madrid. Spain | 19 | 6,2 |
| Hospital Universitario de Araba (Txagorritxu), Alava. Spain | 18 | 5,9 |
| Brigham & Women’s Hospital, Boston, Massachusetts, USA | 11 | 3,6 |
| Hospital Universitari Vall D´Hebron, Barcelona. Spain | 8 | 2,6 |
| Hospital General Universitario de Ciudad Real. Spain | 8 | 2,6 |
| Complexo Hospitalario Universitario de A Coruña. Spain | 8 | 2,6 |
| Hospital das Clinicas da Univerisidade de Sao Paulo, Sao Paulo, Brazil | 7 | 2,3 |
| Municipal Clinical Hospital #17, Moscow, Russia | 7 | 2,3 |
| Stanford University Medical Center, Stanford, California, USA | 6 | 2 |
| Complejo Asistencial Universitario de Salamanca. Spain | 5 | 1,6 |
| Hospital Universitario Puerta Hierro Majadahonda, Madrid. Spain | 5 | 1,6 |
| Hospital Universitario Son Llàtzer, Mallorca. Spain | 4 | 1,3 |
| Yale New Haven Hospital, New Haven, Connecticut, USA | 3 | 1 |
| Hospital Universitario y Politécnico La Fé, Valencia. Spain | 3 | 1 |
| Hospital Clinic, Barcelona. Spain | 3 | 1 |
| Complejo Hospitalario Universitario de Cáceres-San Pedro de Alcántara. Spain | 2 | 0,7 |
| Hospital Universitario de Badajoz. Spain | 2 | 0,7 |
| Hospital Vega Baja, Alicante. Spain | 1 | 0,3 |
| Hospital Universitario Virgen del Rocío, Sevilla. Spain | 1 | 0,3 |
| Azienda Sanitaria Universitaria Giuliana Isontina (ASUGI), Trieste. Italy | 1 | 0,3 |
| Hospital Universiario Virgen de la Victoria, Málaga. Spain | 1 | 0,3 |
| Hospital Universitario Infanta Leonor, Madrid. Spain | 1 | 0,3 |
| Hospital General Universitario de Alicante. Spain | 1 | 0,3 |
| City Hospital #51, Moscow. Russia | 1 | 0,3 |
| Hospital Arquitecto Marcide, Ferrol. Spain | 1 | 0,3 |
| Total | 305 | 100 |
